# Mammography as a method for diagnosing breast cancer

**DOI:** 10.1590/0100-3984.2016.49.6e2

**Published:** 2016

**Authors:** Hilton Koch

**Affiliations:** 1 Full Professor at the Universidade Federal do Rio de Janeiro (UFRJ), Rio de Janeiro, RJ, Brazil. E-mail: hakoch@uol.com.br.

The history of mammography began with Salomon, the German surgeon who, in 1913, studied the application of radiology in breast diseases. The first X-rays of the breasts were taken in 1931 by Romagnoli, who drew attention to the early diagnosis of breast cancer. However, it was Raul Leborgne who, in 1951, published an article on the diagnosis of breast tumors and showed the long cones that adapt an X-ray machine to perform mammograms. Much later, a specific mammography system-the Senograph-was developed^(1)^.

In 1990, the Division of Chronic and Degenerative Diseases of the Brazilian National Ministry of Health decided to launch a campaign for the prevention of breast cancer. For the Colégio Brasileiro de Radiologia (CBR, Brazilian College of Radiology), it was first necessary to know the number of mammographs in Brazil and how those were distributed throughout the country. Although it was determined that there were 585 mammographs, it was also necessary to obtain data on the quality of the equipment and the images produced. Therefore, the CBR Mammography Commission was created. A revolution began, in which the companies began to improve the mammographs and the physicists began to monitor the quality of the mammograms. There was a lack of training for the technicians who performed the examinations.

The greatest achievement was an improvement in the quality of the mammograms. Then came the question of medical interpreters. Despite the efforts of the CBR Mammography Commission, there was no model for the interpretation of mammograms until the appearance of the Breast Imaging Reporting and Data System (BI-RADS), developed by the American College of Radiology. The introduction of the BI-RADS represented a major improvement in the standardization of the language and interpretation of mammograms^(2,3)^. Despite the efforts of the CBR, the Brazilian Federation of the Societies of Obstetrics and Gynecology, and the Brazilian Society of Mastology, the physician interpreters were slow to incorporate the standardization, and processes to correct for errors of interpretation and of practice recommendations began to develop. A project for training professionals in the early detection of breast cancer was developed by the Laboratory of Radiological Sciences at the State University of Rio de Janeiro, through a course offered to physician mammogram interpreters in the state of Rio de Janeiro. Pre-and post-tests showed that the result was unsatisfactory, because there was no improvement in the description of the breast changes, as well as because there was a lack of consistency between the final BI-RADS rankings and the practices recommended.

From time to time, someone proposes a project to implement a program for the early detection of breast cancer. On several occasions, there was discussion regarding whether the ideal age for women to undergo their first mammography examination would be 40 years or 50 years. According to the Brazilian Society of Mastology, the ideal is 40 years of age, whereas the Brazilian National Ministry of Health defines it as 50 years of age.

What is the cost to the Brazilian government for performing mammograms via the Unified Health Care System? Such a practice will help a segment of the female population who will benefit from early diagnosis. Another problem is the treatment-is there sufficient availability of resources, equipment, and personnel? Analyzing the results of the study conducted by Freitas-Junior et al.^(4)^, published in this issue of **Radiologia Brasileira**, one can observe that, in the first assessment of the ratio between the number of mammographs and the number of women eligible to undergo mammography, in the 1990s, 75% of mammographs were located in the southern and southeastern regions of Brazil, where there was one mammograph for every 23,000 women, compared with one for every 90,000 women in the northeastern region of the country. Although there has been a significant increase in the mammograph/woman ratio, it has not yet had the expected effect on mass screening, despite the increase in demand for mammograms. Over time, it will always be necessary to review the quality of the images obtained^(5)^, as there will always be a need for the continuing education of the interpreters.
